# Repeated neonaticide: differences and similarities to single neonaticide events

**DOI:** 10.1007/s00737-018-0850-1

**Published:** 2018-05-23

**Authors:** Claudia M. Klier, Sabine Amon, Hanna Putkonen, Paula Fernandez Arias, Ghitta Weizmann-Henelius

**Affiliations:** 1 Department of Pediatrics and Adolescent Medicine, Medical University of Vienna, Vienna, Austria; 2 Department of Psychology, University of Vienna, Vienna, Austria; 3 Vanha Vaasa Hospital, Vaasa, Finland; 4 Addiction Psychiatry, Helsinki University Central Hospital, Helsinki, Finland; 5 Public Policy Research Centre, Universidad Mayor, Santiago, Chile; 6 Monash Deakin Filicide Research Hub, Department of Social Work, Monash University, Melbourne, Australia; 7 Department of Psychology and Logopedics, Åbo Akademi University, Turku, Finland

**Keywords:** Filicide, Child murder, Neonaticide, Repeated neonaticide

## Abstract

This study aims to identify differences between single and repeat perpetrators of filicide by using register-based data. The study used register-based, comprehensive, nationwide data from both Austria and Finland. The current study covers 23 perpetrators, 20 single and 3 repeat perpetrators, with a total of 28 victims. All victims had a maximum age of 24 h and all perpetrators were women. Every third victim of neonaticide was a victim of a repeat case. The repeat perpetrators were older; had a higher number of children over their lifespan, some of whom lived with them; were more likely to live within established family structures; had higher levels of education and employment; had a higher proportion of personality disorders; and were more likely to identify stress factors during pregnancy. One unexpected finding was low levels of awareness about pregnancy within the perpetrator’s circle remain a risk factor, especially for repeat perpetrators. Arguably, the quality of interpersonal relationships these women have may be affected by their own mental health issues and life experience and vice versa.

## Introduction

Neonaticide is regarded as a crime of low prevalence but given its characteristics—there is no birth certificate, no one else may be aware of the child’s existence, and thus there is no “missing person”—it remains hidden. Due to these difficulties, few publications have been rigorous enough to come up with incidence rates (Klier et al. 2013; Overpeck et al. 2002; Putkonen et al. 2007; Tanaka et al. 2017; Tursz and Cook 2011).

Early research demonstrated that neonaticide perpetrators differ from other filicide perpetrators regarding motives and individual traits (Resnick 1970). However, the group of women who commit neonaticide show common characteristics (Amon et al. 2012; Putkonen et al. 2007; Vellut et al. 2013). As filicide, and particularly neonaticide, are inconceivable crimes and difficult for the public to understand, cases involving mothers killing more than one newborn over time are even harder to comprehend.

In 2016, our group published a new classification of filicide (Putkonen et al. 2016) where neonaticides represented a specific subgroup: the infanticidal mothers. As a group, they were the youngest (mean age 27 years, SD = 7.3, range 16–42). The members of this subgroup had almost no previous dealings with the authorities. The most common motive for the neonaticide was the child being unwanted (26.8%) and there were no cases of psychotic or extended suicide motivation. Regarding the killing method, suffocation (36%) was the most common.

Inside this neonaticide subgroup, one out of three was a repeat case. In our new typology of filicide, both single and repeat neonaticides were clustered in the same group. But do these repeat perpetrators differ from the single perpetrators? In this study, we would like to shed light on the differences and similarities of single vs. repeat neonaticides.

## Material and methods

### Data collection

The present study uses register-based, comprehensive, nationwide data from both Austria and Finland. Merging data across jurisdictions and/or countries is greatly facilitated if uniform data collection procedures are in place. We were able to combine data from Finland and Austria because the countries have similar legal traditions and social systems (Bernitz 2010). In our previous studies, we found the two countries to be sufficiently similar in their population demographics and organizations to allow parallel data gathering without significant distortions from cultural or systemic differences (Putkonen et al. 2009). The study covered all recorded filicide cases between 1995 and 2005, inclusive. The operative definition of filicide was the killing of a child by their parent; parent was defined as biological, step, or foster parent. To be classified as a step-parent, an authentic parental relationship, defined as a longstanding, live-in relationship, had had to exist. There were 23 neonaticide perpetrators, i.e., women who killed newborns within their first 24 h of life.

Data on children who died younger than 18 years were collected from coroner reports and death certificates from the Coroner Institutions of Austria and from Statistics Finland, respectively. In total, there were 238 child homicide victims, 152 (86 Austria, 66 Finland) of whom were filicide victims. The number of perpetrators was 124; 79 females and 45 males. All key national results and detailed information on data gathering have been reported in our previous work (Putkonen et al. 2009). In addition to the Austrian coroner reports, all the court files from the Austrian Department of Justice were studied, so as to include all information pertinent to the present study. Similarly, in Finland, in addition to the death certificates, other registers were examined: police files, the National Finnish Hospital Discharge Register, forensic psychiatric examination reports from registers of the National Institute for Health and Welfare, and criminal records from the Legal Register Centre. To sum up, all relevant register-based data were collected on all known filicide cases in both countries during the period in question: 1995–2005.

### Variables

The variables in the present study were chosen based on an extensive review of previous literature and a preliminary analysis of the data. We have previously published work on this material (Amon et al. 2012; Putkonen et al. 2010; Putkonen et al. 2011), and our earlier results indicated which variables would prove valuable. Not all registers provide data on all variables for all perpetrators. None of the cases were excluded, but variables with many missing values were eliminated from the analyses. The variables were coded according to a structured list based on previous literature.

The variables used in the final analyses covered the perpetrator’s history, that is, age, gender, socioeconomic background (marital status, education, employment history), possible criminal record, and mental health information (childhood conduct disorder features as defined by DSM-IV (American Psychiatric Association 1994), as well as variables related to the circumstances existing before and during the crime. Pre-offense circumstances included information on whether there were any prior dealings with authorities (police, health care services, school, pre-school, or child welfare authorities). It also covered any record of domestic violence, that is, physical violence directed at any member of the current family unit, not only a child. The stability, or lack thereof, of the parental relationship was also a variable of interest.

The variables connected with the circumstances existing during the crime included whether the offender was intoxicated with alcohol or drugs at the time of the offense, the age of the victim(s), defined as infant (less than one year old) or not, and the killing methods.

### Inter-rater agreement

Three researchers rated the data, one in Austria (SA) and two in Finland (GWH, HP). Owing to the nature of the phenomenon under study, it was impossible to code the variables blind to the gender of the offender. Moreover, for linguistic and practical reasons, inter-rater agreement could not be assessed using cases included in the present study; the original documents were in German or Finnish and were thus accessible only to the Austrian and Finnish raters, respectively. The documents could not be translated into English because confidentiality considerations prevented the transportation of the records outside the country of origin. Therefore, in order to assess inter-rater agreement, all three raters examined two cases from the UK and the calculation was made using Cohen’s kappa *κ* (Wirtz and Caspar 2002). Only variables on which the raters were in substantial or perfect agreement were included in the study. Kappa (*k* = 1.0–0.75) was excellent on all variables used in the present study (Cicchetti 1994; Cicchetti 2001). In Finland, kappa was also calculated on actual cases included in the study, because there were two Finnish raters. The agreement was excellent on all variables used (*k* = 1.0–0.80). Prior to rating the actual cases which formed our source material for this study, the raters practiced and discussed the issues of rating extensively, to ensure uniform coding.

### Data description

The current study covers 23 perpetrators with a total of 28 victims. All victims had a maximum age of 24 h and all perpetrators were women. We have previously reported the individual prevalence of all the variables in this study (Putkonen et al. 2009; Putkonen et al. 2010; Putkonen et al. 2011).

Out of 124 filicide cases, 28 (23%) were classified as neonaticide. There was a significant difference in the neonaticide rate (Putkonen et al. 2009), 23 cases in Austria and 5 cases in Finland or 2.59 and 0.8 per 100,000 live births, respectively (Statistic Austria 2009; Statistic Finland 2011). Repeat neonaticides were observed only in Austria.

## Results

Within the neonaticide subgroup, there were 3 (13%) repeat perpetrators who were responsible for 8 (29%) cases (2–2-4 victims respectively) and 20 (87%) single perpetrators with 1 (71%) case each.

### Age

The difference of the average age at the time of index delivery between single [mean = 26.5, SD = 7.7] and repeat [mea*n* = 32.87, SD = 5.7] perpetrators was significant.

### Education and employment

The repeat perpetrators had a significant [chi-square: *Χ*^2^ = 9.707; *df* = 2, *p* = 0.008] higher level of education than single ones; all (*n* = 3) of them had a medium/secondary school level vs. seven (35%) of the single perpetrators [11 (55%) compulsory school, 2 (10%) no finished school]. Regarding the employment status (each pregnancy was considered to account for the possibility of social mobility between pregnancies), in six cases (75%) of the repeat pregnancies, the mothers were employed vs. ten (50%) of the single ones, in two cases (25%) of the repeat pregnancies mothers were unemployed vs. eight cases (40%) of the single ones, and none of the repeat perpetrators were a student vs. two (10%) of the single ones.

### Number of children

The 23 neonaticide perpetrators gave birth to 49 children (28 neonaticides and 21 alive children). Ten (48%) out of 21 alive children did not live with their biological mothers; those 10 children were born to 5 single neonaticide perpetrators.

Repeat perpetrators had a significantly higher number of biological children (neonaticide victims included) [mean = 4.33, SD = 0.58] compared to single perpetrators [mean = 1.8, SD = 1.15] (see Table 1).

**Table 1:** Victims and alive children of perpetrator

	Repeat (*n* = 3)	Single (*n* = 20)
Number of children^a^	13	36
Victims	8	20
Alive children	5	16
Alive children in fulltime care of perpetrator	5	6
Child-mother rate	1.7	0.3

^a^*T* test: *df* = 21, *p* = 0.001

### Relationship status

In 16 (57%) out of 28 of the pregnancies, the neonaticidal mother had a relationship with the victim’s father (married or common law: repeat (5/8) 63%, single (8/20) 40%; risk of separation or divorce: repeat (1/8) 13%, single (2/20) 10%). The average length of the relationship before pregnancy was 3 years (mea*n* = 3.2 years, SD = 1.95). The study found there was a significant difference in the living status during pregnancy of repeat and single perpetrators [chi-square: *Χ*^2^ = 113.913, *df* = 5, *p* = 0.016].

None of the repeat perpetrator’s existing partners reported they had knowledge of the pregnancies, although they had sexual relationships during the pregnancies. Only 3 (15%) out of 20 of the single perpetrator’s partners reported knowledge of the pregnancy.

### Contraception and pregnancy variables

Contraception use was found in 14 (50%) out of 28 pregnancies [repeat (5/8) 63% vs. single (9/20) 45%]: At six (21%), pregnancies hormonal contraception or IUD were used [repeat 0 vs. single (6/20) 30%] and at seven pregnancies (25%) a condom [repeat (4/8) 50% vs. single (3/20) 15%]. Some physical symptoms of pregnancy were present in most cases, but this presence varied: 20 (71%) pregnancies weight gain [repeat (4/8) 50% vs. single (16/20) 80%] and 15 (54%) pregnancies amenorrhea [repeat (1/8) 13% vs. single (14/20) 70%].

### Awareness about pregnancy

In 8 (29%) out of 28 of the pregnancies, nobody in the social environment ever asked about a pregnancy [repeat (6/8) 75% vs. single (2/20) 10%]. In 20 (71%) out of 28 of the pregnancies, the perpetrators were asked only once, if they were pregnant, but successfully negated it [repeat (2/8) 25% vs. single (13/20) 65%].

The main motive for negating the pregnancy (reported only in 26 cases) was fear of abandonment and negative response from others (58%) [repeat (6/8) 75% vs. single (9/18) 50%] and no motive was found in four cases [repeat 0 vs. single (4/18) 22%].

### Stress factors during pregnancy

Some stress-related factors, adapted from DSM-IV, were identified as present during the 28 individual pregnancies: problems with primary support group [repeat (4/8) 50% vs. single (5/20) 25%], occupational problems [repeat (3/8) 38% vs. single (3/20) 15%], economic problems [repeat (6/8) 75% vs. single (6/20) 30%], and no problems [repeat 0 vs. single (10/20) 50%].

### Delivery

In all 28 cases, the delivery was unassisted and took place in the perpetrator’s home. In 10 cases [all of them single], other people were present in the house but had no awareness of the delivery taking place.

### Psychiatric evaluation

Following the neonaticide, 18 (78%) out of 23 women underwent a forensic psychiatric examination, 3 (13%) did not, and 2 (9%) (one repeat and one single perpetrator) died immediately after delivery. Mood disorders were diagnosed only within single perpetrators [(5/16) 31%]. All alive repeat perpetrators had a personality disorder vs. 4 (25%) out of 16 of the single ones, a brief psychotic disorder was diagnosed for 2 (13%) out of 16 of the single and none for the repeat perpetrators. No diagnosis could be found for 5 (31%) out of 16 of the single perpetrators.

Considering childhood trauma, 11 (48%) out of 23 of perpetrators had traumatic experiences: two out of three repeat perpetrators and 9 (45%) out of 20 single perpetrators. Repeated trauma in adulthood was also identified in 1 out of 3 repeat neonaticide perpetrators and in 3 (15%) out of 20 single neonaticide perpetrators.

## Discussion

This is the first study to use a register-based sample to compare cases of single to repeat neonaticides. Even though neonaticides are rare events every third case of neonaticide was a repeat case. Although the group of repeat neonaticides was very small, consisting of three women, the comparison allows a window into some interesting differences.

Women in the repeat group were older than single perpetrators. The older age is to be expected as repeat perpetrators have more pregnancies over their lifespan. Consequently, when they are detected, they are already older compared to their single neonaticidal counterparts. The study also found that repeat perpetrators had a higher number of biological children including neonaticide victims. This is an interesting finding given the characterization of neonaticidal mothers as young and without children (Resnick 1970). The greater number of biological children was also described in our previous study on circumstances of pregnancy where we found a high level of fecundity in women, but in that study, many of those children were not living with the perpetrator (Amon et al. 2012). In this study, we found that more children live under the care of repeat perpetrators.

The high rate of contraception use reported in the repeat pregnancies is somewhat puzzling. Arguably, if women have a consistent contraceptive behavior, the fertility rate should not be so high. It might be that women reported more contraceptive behavior than they performed or that the contraceptive method failed. Justad-Berg et al. (2015) found that many women with recurring unwanted pregnancies who sought out terminations have children and/or used contraception at the time of conception. The findings of this Norwegian group suggest that high fecundity is an underlying precursor for repeated pregnancy termination and other risks related to an unwanted pregnancy.

Repeat neonaticide perpetrators reported a low rate of amenorrhea during pregnancy, a perplexing finding. Repeat neonaticidal mothers reported even less awareness of body changes than their single counterparts. On the other hand, they had more pregnancies and might have been more experienced in recognizing their pregnancy but did not report the changes they did notice. This finding coupled with the high rate of partnerships found among both groups raises questions about the environment and the quality of the relationships perpetrators have.

A relationship with the father was present in more than half of all pregnancies (40% single and 63% repeat perpetrators); however, a third of the women were not asked about it and 71% negated their pregnancy, some despite the presence of physical changes. The difference in living arrangements between single and repeat perpetrators was also found to be significant. Repeat perpetrators were found to live in established family systems. It could be hypothesized that the quality of the relationships is questionable. None of the repeat partners reported knowledge of the pregnancy although most had sexual contact and lived with the mother (Putkonen et al. 2007).

The women’s inability to bring up the subject may also be related to personality disorders, as well as to fear of detection of the previous child births. However, the number of neonaticides has decreased in Finland since our prior report (Putkonen et al. 2007). The explanation for this is the changes in attitudes regarding premarital relationships and pregnancies, which now are as accepted as pregnancies in conjugal families. Seemingly no talks about long-term family planning took place, although some had children in their care and there were economic problems.

Social unawareness is a phenomenon which Amon et al. (2012) specifically focused on and identified as more relevant than any other psychosocial variable. This finding is further reinforced by this new study where it seems the social environment was more ignorant about the expectant mother as she went thru pregnancy several times without anyone recognizing her condition. The cases in the literature of repeated neonaticide describe this phenomenon, but no study up to now has employed a register-based sample (Burton and Dalby 2012; Funayama et al. 1994; Funayama and Sagisaka 1988; Heitzman et al. 2013). However, it is important to note that social unawareness was not found to be a significant difference between single and repeat but rather a risk factor for all neonaticides. The most important risk factor, social unawareness, did not differ between perpetrator groups.

Even though repeat perpetrators had a higher level of education, they were more susceptible to identifying economic stress factors during pregnancy. They were also more likely to identify problems with their primary support group. Generally, women who commit neonaticide have a high load of problems in childhood such as trauma and abuse (Amon et al. 2012); in consequence, their educational pathway is compromised. Given this does not seem to be the case for repeat perpetrators, it may be possible to argue that their tolerance for stressors is lower given their higher achievements in life.

The diagnosis of personality disorder is present in all alive repeat perpetrators but only in one quarter of the single ones. This is an interesting finding as the repeat group could differ in respect to the psychopathology but this fact has never been addressed in a register-based study. Regarding the personality diagnosis, which determines potential problems with qualitative social binding, it seems these women had to find a solution by themselves for the anticipated problems of the expected child, because of the perceived inability to talk with others specially their partners.

## Limitations

This research was an initial study comparing single vs. repeat neonaticide cases. It can only show trends of differences, because of the small numbers of repeat offenders.

## Conclusion

This study argues that repeat perpetrators of neonaticide seem to differ from their single counterparts. The average age of the three repeat perpetrators was higher due to the length of time over which the neonaticides were committed and they have more children over their lifespan. These children tend to live with them, and added to the higher presence of partners, it is clear that they live in established family systems. Repeat perpetrators reported a higher rate of contraceptive use but less physical symptoms of pregnancy were recognized by them and their families. It is possible to speculate that their negation of pregnancy comes from the potential abandonment they fear once the pregnancy is known. Repeat perpetrators also have a higher rate of diagnosed personality disorders which can impact the quality of their relationships and their ability to communicate with their partners.

One striking similarity is the high risk that social unawareness poses for both groups even though it might be difficult to understand how this was possible with repeat perpetrators. Overall, it is possible to say that there seem to be differences that distinguish repeat perpetrators from their single neonaticidal peers but also that repeat perpetrators are different from the single mothers that have been discussed in previous research on neonaticide. Future research should focus on the quality of the relationships these women are able to establish and examine their ability to establish social bonds.
